# Peripheral vestibular syndrome in cats: Clinical presentation, diagnostic findings and outcome in 196 cases

**DOI:** 10.1002/vetr.5324

**Published:** 2025-05-20

**Authors:** Jordina Caldero Carrete, Steven De Decker, Holger A. Volk, Rodrigo Gutierrez‐Quintana, Anna Morgana Mosel, Rita Gonçalves

**Affiliations:** ^1^ Small Animal Teaching Hospital, School of Veterinary Science University of Liverpool Neston UK; ^2^ Queen Mother Hospital for Animals Royal Veterinary College Hatfield UK; ^3^ Department of Small Animal Medicine and Surgery University of Veterinary Medicine Hannover Hanover Germany; ^4^ School of Biodiversity, One Health and Veterinary Medicine, College of Medical Veterinary and Life Sciences University of Glasgow Glasgow UK

## Abstract

**Background:**

Disorders of the vestibular system are frequent in cats. This study aimed to describe the clinical presentation, diagnostic findings, underlying aetiologies and outcome of cats with peripheral vestibular syndrome (PVS).

**Methods:**

This was a retrospective study of cats presented with PVS at four referral hospitals. All of the cats underwent magnetic resonance imaging or computed tomography of the head. Multivariable logistic regression analysis was performed to identify clinical variables associated with the most common diagnoses.

**Results:**

A total of 196 cats were included. The most common diagnosis was otitis media/interna (OMI) alone (*n* = 91) or with aural polyps (*n* = 49), followed by idiopathic vestibular syndrome (IVS) (*n* = 47), middle ear neoplasia (*n* = 7) and congenital vestibular syndrome (*n* = 2). A diagnosis of OMI was associated with younger age (odds ratio [OR] = 0.993, 95% confidence interval [CI]: 0.986‒1.000, *p* = 0.044), longer duration of clinical signs (OR = 1.034, 95% CI: 1.008‒1.061, *p* = 0.009), history of otitis externa/upper respiratory signs (OR = 5.245, 95% CI: 1.849‒14.882, *p* = 0.002), facial nerve paralysis (OR = 6.531, 95% CI: 1.287‒31.335, *p* = 0.023) and Horner syndrome (OR = 15.804, 95% CI: 2.014‒124.02, *p* = 0.009). Follow‐up data for 104 cats revealed full recovery in 33 cats, partial recovery in 67 cats and no recovery in four cats.

**Limitations:**

The limitations of this study include its retrospective nature, multicentre approach and incomplete outcome data.

**Conclusion:**

OMI is the most common cause of PVS in cats and is associated with younger age, longer duration of clinical signs, history of otitis externa/upper respiratory signs, facial nerve paralysis and Horner syndrome. The majority of cats diagnosed with OMI and IVS experience at least partial recovery from the vestibular signs.

## INTRODUCTION

The vestibular system is the primary sensory system that maintains body posture, balance and normal orientation relative to the influence of gravitational forces.^1^, ^2^ The anatomical portions of the vestibular system can be divided into peripheral and central components. The peripheral components of the vestibular system are located in the inner ear and include the vestibular receptors and vestibular division of the cranial nerve VIII, while the central components are the vestibular nuclei in the medulla oblongata and the flocculonodular lobes and fastigial nucleus of the cerebellum.^3^, ^4^, ^5^

Disorders of the vestibular system occur frequently in cats.^6^ The main causes include otitis media/interna (OMI), idiopathic vestibular syndrome (IVS) and middle ear neoplasia.^7^, ^8^, ^9^, ^10^, ^11^ In cats, OMI is often associated with aural polyps that arise from the mucosa of the tympanic cavity or the auditory tube.^11^, ^12^, ^13^, ^14^, ^15^ Other causes of peripheral vestibular signs include ototoxicity, trauma to the tympanic bulla or petrosal portion of the temporal bone and congenital vestibular syndrome.^4^, ^6^, ^7^, ^9^, ^16^, ^17^

Peripheral vestibular dysfunction results from disorders involving the vestibular receptor or nerve.^12^ Clinical signs of unilateral peripheral vestibular dysfunction include asymmetric ataxia and loss of balance, with leaning, falling and rolling towards the side of the lesion, spontaneous or positional horizontal or rotatory jerk nystagmus, head tilt and positional strabismus.^2^, ^3^, ^12^ Ipsilateral Horner syndrome and facial neuropathy may be present in OMI due to the postganglionic sympathetic fibres and the facial nerve passing through the tympanic cavity.^1^, ^2^, ^17^

The largest previous studies investigating vestibular dysfunction in cats included a combination of feline populations with central and peripheral vestibular dysfunction.^7^, ^9^ OMI and IVS have been described as the most commonly reported underlying aetiologies for peripheral vestibular syndrome (PVS).^7^, ^9^ However, outcome information is sparse, and the outcome of these patients remains poorly documented.^7^, ^11^ Therefore, this study aimed to determine the prevalence of the different causes of PVS in cats, describe the clinical presentation, diagnostic findings and outcome and identify possible risk factors associated with the underlying aetiologies.

## MATERIALS AND METHODS

The digital medical records of the Small Animal Hospitals of the University of Liverpool, Royal Veterinary College, University of Glasgow and University of Veterinary Medicine Hannover were searched to identify cats with a neurolocalisation compatible with peripheral vestibular dysfunction and a final diagnosis affecting a structure within the peripheral vestibular system that underwent magnetic resonance imaging (MRI) or computed tomography (CT) of the head as part of their diagnostic investigations between 2009 and 2023. The search terms used were ‘feline’ and ‘vestibular’ or their variations, with only cases meeting the inclusion criteria being reviewed. A clinical presentation suggestive of unilateral PVS was defined by observing one or more of the following signs: asymmetric ataxia, spontaneous or positional nystagmus, head tilt and/or positional strabismus. In cases with ataxia alone, the absence of detectable paresis or proprioceptive deficits was required to differentiate from proprioceptive ataxia, and cases were included if they presented leaning and falling to one side. Evidence of facial nerve paralysis or Horner syndrome (miosis, ptosis, enophthalmos and protrusion of the third eyelid) was also recorded when present. Bilateral vestibular dysfunction was defined on the basis of observing a symmetric ataxia and oscillating head movements, with the absence of a head tilt and absence of pathological or physiological nystagmus.^1^, ^4^ Cats with mentation changes, postural deficits and/or deficits associated with additional cranial nerve deficits (other than cranial nerves VII and VIII) were diagnosed with suspected central vestibular dysfunction^12^ and therefore excluded from the study. Cats were also excluded if their medical records were incomplete, if they had not undergone MRI or CT of the head or if they had signs consistent with peripheral vestibular dysfunction but advanced imaging findings indicating central vestibular involvement (except those with otogenic intracranial extension without clinical signs of central vestibular disease).

The following data were extracted from the medical records of cats meeting the inclusion criteria: signalment (age, sex, neuter status, bodyweight and breed); previous medical history (previous vestibular episodes, concurrent diseases and initiation of medical therapy before referral); onset, progression and duration of clinical signs; physical and neurological examination findings on admission; diagnostic testing findings, including results of advanced imaging, cerebrospinal fluid (CSF) analysis, histology and other ancillary tests; and treatment and outcome where available. Breed was categorised as purebred or non‐purebred for analysis. Onset of the clinical signs was defined as the period of time between when the cat was last clinically normal and the most severe neurological status and was classified as acute (24 hours or less) or chronic (more than 24 hours). Progression was divided into progressing, non‐progressing (including stable and improving) and waxing and waning. Treatment was categorised into four groups: no treatment, medical treatment only, myringotomy/ear flush with medical treatment, and surgery (bulla osteotomies and traction avulsion) with or without medical treatment.

CT was performed with either a 16‐slice (PQ 500, Universal Systems, Solon; GE Healthcare), an 80‐slice (Aquilion Prime, Canon), a 256‐slice (IQon Spectral CT, Philips Healthcare), a 320‐slice (Canon Aquilion ONE/GENESIS; Toshiba Medical Systems) or a dual‐slice (Dual Slice Somaton Spirit, Siemens) helical scanner under sedation or general anaesthesia. Injectable ioversol (600 mg/kg) was administered intravenously (IV). MRI was performed with a 1.5 T (Phillips Interna 1.5 T system, Philips Medical Systems), a 1.5 T (Gyroscan ACS‐NT, Philips Medical System), a 1 T (Magnetom Harmony, Siemens) or a 3.0 T (Phillips Achieva, Philips Medical Systems) scanner under general anaesthesia. The following sequences were obtained in all patients: turbo spin‐echo T2‐weighted images (T2W) in transverse, sagittal and dorsal planes, T2W fluid‐attenuated inversion recovery (FLAIR) and pre‐ and postcontrast (IV injection of 0.1 mmol/kg of gadopentetate dimeglumine) T1‐weighted images (T1W) in transverse plane. Thin slice sequences (T1W gradient echo VIBE‐MRI sequences—1 mm slice thickness; T2W gradient echo CISS, BALT‐GRAD or T1W mDIXON—2 mm slice thickness) postcontrast were also obtained in most but not all patients. One of each institution's board‐certified radiologists reviewed the MRI and CT images from their respective institution.

CSF was collected from the cerebellomedullary cistern under general anaesthesia. For CSF analysis, pleocytosis was defined as a total nucleated cell concentration (TNCC) of more than 5 cells/µL, and total proteins (TP) were considered elevated when greater than 0.25 g/L.^18^

Short‐term outcome information (less than 6‐month follow‐up period) was obtained from the medical records. Long‐term outcome information (follow‐up period longer than 6 months) was evaluated when available and was obtained through the medical records when this was possible or via telephone consultation with the referring veterinarian or owner. When short‐ and long‐term outcomes were available for the same case, only the long‐term outcome was used as follow‐up information. Conforming to local ethics, only owners of cats that were still alive at the time of data collection were contacted. Recurrence was defined as the return of peripheral vestibular signs. Time to episode recurrence was divided into less than 3 months, within 3‒12 months and later than 12 months after initial diagnosis. Full recovery was defined as complete resolution of neurological signs, partial recovery as an improvement in signs but persistence of some neurological deficits and no recovery as persistence of the presenting neurological deficits.

A diagnosis of OMI was achieved when advanced imaging showed compatible changes and was supported by cytology and/or culture.^19^, ^20^, ^21^ Findings consistent with OMI on MRI were hyperintense tympanic bulla material on T2W images, with or without peripheral contrast enhancement on T1W images, and lack of signal on T2W images and/or increased signal intensity on FLAIR images from the intralabyrinthine fluid with or without contrast enhancement on T1W images.^22^, ^23^, ^24^ CT changes consistent with OMI included thickening, irregularity or lysis of the tympanic bulla wall, soft tissue material within the tympanic bulla with or without contrast enhancement, and destruction of the inner ear.^25^, ^26^, ^27^ In both imaging modalities, aural polyps were suspected when well‐defined homogeneous pedunculated growths with postcontrast rim enhancement were found invading the tympanic bulla.^14^, ^28^, ^29^, ^30^ In cases where histology could not be performed, a polyp was suspected based on imaging findings only. Neoplasia was diagnosed when an abnormal soft tissue mass affecting the middle ear was identified on MRI or CT and further confirmed by histopathology.^22^, ^25^, ^27^ Cats were diagnosed with IVS when advanced imaging did not show any abnormalities or only identified facial and/or vestibulocochlear nerve changes on MRI.^3^, ^6^, ^31^, ^32^, ^33^ A diagnosis of congenital vestibular syndrome was considered when vestibular signs developed at or shortly after birth and there were no abnormalities on advanced imaging.^3^, ^4^, ^6^

Statistical analysis was performed using the software SPSS 27.0 (SPSS). Continuous data were tested for normality using the Shapiro‒Wilk test. Most data were not normally distributed, so descriptive statistics were calculated as medians and interquartile ranges (IQRs). Univariable logistic regression was performed to identify variables associated with final diagnosis (only cats diagnosed with IVS and OMI were included in this analysis). Factors hypothesised to be associated with these diagnoses were age, bodyweight, breed (purebred or non‐purebred), centre, onset of the clinical signs (acute or chronic), progression or not of the clinical signs, duration of the clinical signs (days), presence of previous vestibular episodes, previous history of otitis externa or upper respiratory signs and neurological signs (head tilt, nystagmus, facial nerve paralysis and Horner syndrome). Before multivariable analysis, all variables were assessed for correlation using Spearman's rank correlation coefficients. If Spearman's rank correlation coefficient was more than 0.8, the most statistically significant or biologically plausible variable was selected. Any independent variable demonstrating some association on preliminary univariable analysis (*p* < 0.25) was considered for inclusion in a multivariable model. Multivariable models were constructed with a manual backwards stepwise removal approach where variables with a *p*‐value of less than 0.05 were retained. The association between outcome and the presence or absence of intracranial extension in cats with OMI was evaluated using a chi‐squared test in a post hoc analysis.

## RESULTS

A total of 196 cats fulfilled the inclusion criteria (76 of which were included in a previous study^9^). Non‐purebred cats were more common (*n* = 131, 66.8%), consisting of domestic longhair (*n* = 11) and domestic shorthair (*n* = 120). The most prevalent purebred cats were British shorthair (*n* = 14), Maine coon (*n* = 12), Bengal (*n* = 7), Burmese (*n* = 6) and Siamese (*n* = 6). The study population consisted of 107 males (54.6%, 98 neutered) and 89 females (45.4%, 83 neutered). The median age at diagnosis was 96 months (interquartile range [IQR] 48‒144), and the median bodyweight was 4 kg (IQR 3.5‒4.6). Thirty‐four cats (17%) were reported to have had at least one previous episode of vestibular dysfunction. Previous history of otitis externa and/or upper respiratory tract disease was reported in 68 cats (34%). Thirty‐six cats (18.4%) presented with an acute onset of clinical signs, and 160 (81.6%) presented with a chronic onset. In 130 cats (66.3%), medications had been administered for their PVS signs prior to referral. The median duration of clinical signs prior to presentation was 7 days (IQR 2‒30). Clinical signs were non‐progressive in 103 cats (52.6%), progressive in 50 cats (25.5%) and waxing and waning in 43 cats (21.9%). Clinical signs on presentation included vestibular ataxia (*n* = 162, 82.7%), head tilt (*n* = 152, 77.6%), nystagmus (*n* = 82, 41.8%), Horner syndrome (*n* = 37, 18.9%), oscillating head movements (*n* = 26, 13.3%), facial nerve paralysis (*n* = 26, 13.3%) and positional strabismus (*n* = 12, 6.1%). Seven cats presented with vestibular ataxia as the only clinical sign, of which three had a previous diagnosis of otitis externa and/or media, three had progressive signs and one had recurring episodes of balance loss (and was always assessed after improvement). Bilateral peripheral vestibular signs were present in 26 cats (13.3%). The clinical information is summarised in Table 1.

**TABLE 1:** Signalment, clinical presentation, neurological examination and diagnostic findings of 196 cats with peripheral vestibular syndrome

	Number of cats	Median age (months)	Sex	Onset	Median duration of signs (days)	Progression	Head tilt	Bilateral head excursion	Ataxia	Nystagmus	Facial nerve paralysis	Horner syndrome	Abnormalities in CN VII/CN VIII in MRI	CSF abnormalities
OMI	140 (71.4%)	84	M: 78 F: 62	Acute: 21 Chronic: 119	8	Prog: 41 Non‐prog: 66 Waxing and waning: 33	116 (82.82%)	19 (13.6%)	111 (79.3%)	53 (79.3%)	24 (17.1%)	35 (25%)	CV VII: 14 (10%) CN VIII: 18 (12.8%)	Pleocytosis: 7 (5%) Pleocytosis and increased TP: 4 (2.8%)
IVS	47 (24%)	117	M: 24 F: 23	Acute: 23 Chronic: 14	3	Prog: 8 Non‐prog: 30 Waxing and waning: 9	32 (68%)	6 (12.7%)	43 (91.4%)	26 (55.3%)	2 (4.2%)	1 (2.1%)	CN VIII: 3 (6.38%)	Pleocytosis: 2 (4.2%) Ad: 1 (2.1%)
Neoplasia	7 (3.6%)	111	M: 4 F: 3	Acute: 2 Chronic: 5	14	Prog: 1 Non‐prog: 5 Waxing and waning: 1	3 (42.8%)	0	6 (85.7%)	3 (42.8%)	0	1 (14.2%)	0	0
Congenital vestibular syndrome	2 (1%)	8.5	M: 1 F: 1	Acute: 0 Chronic: 2	2432	Prog: 0 Non‐prog: 2 Waxing and waning: 0	0	1 (50%)	2 (100%)	0	0	0	0	0

Abbreviations: Ad, albuminocytological dissociation; CN, cranial nerve; CSF, cerebrospinal fluid; F, female; IVS, idiopathic vestibular syndrome; M, male; Non‐prog, non‐progressive; OMI, otitis media/interna; Prog, progressive; TP, total protein.

The most commonly diagnosed condition was OMI (*n* = 140, 71.4%), alone (*n* = 91) or secondary to polyps (*n* = 49), followed by IVS (*n* = 47, 24%), middle ear neoplasia (*n* = 7, 3.6%) and congenital vestibular syndrome (*n* = 2, 1%). The most common neoplasia was middle ear adenoma (*n* = 3), followed by adenocarcinoma (*n* = 2), endocrine tumour (*n* = 1) and squamous cell carcinoma (*n* = 1).

**FIGURE 1:**
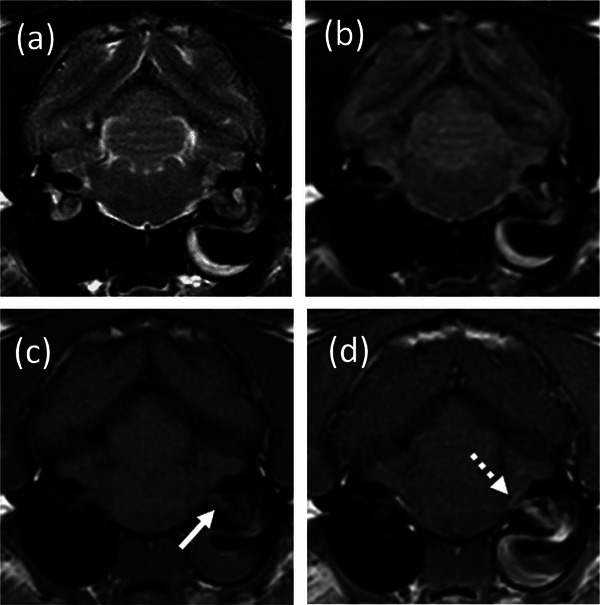
Transverse (a) T2‐weighted (T2W), (b) T2W fluid‐attenuated inversion recovery (FLAIR), (c) T1‐weighted (T1W) pre‐gadolinium contrast and (d) T1W post‐gadolinium contrast administration magnetic resonance images at the level of the tympanic bullae in a cat with an aural polyp and otitis media/interna. There is T2W/FLAIR hypo to isointense and T1W hypointense material filling part of the ventromedial compartment of the left tympanic bullae, surrounded by T2W/FLAIR hyperintense and T1W isointense material. The left cochlea has a mild loss of T2W signal and does not suppress on FLAIR. Postcontrast images show moderate heterogeneous enhancement of the material, bulla lining and cochlea. The left facial and vestibulocochlear nerves are mildly thickened (c, arrow) and contrast enhancing. There is mild and focal meningeal enhancement (d, dotted arrow)

**FIGURE 2:**
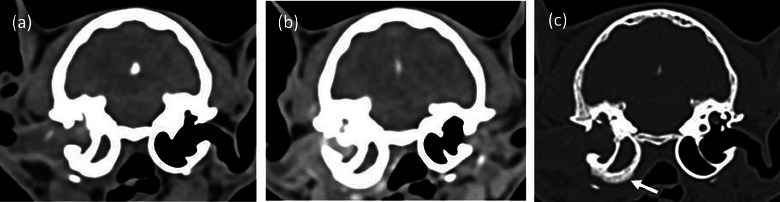
Transverse (a) soft tissue window, (b) soft tissue window post‐ioversol contrast administration and (c) bone window computed tomography images at the level of the tympanic bullae in a cat with an aural polyp and otitis media/interna. There is a large volume of soft tissue/fluid attenuating and contrast‐enhancing material in the right tympanic bulla, which extends into the external ear canal. Note the periosteal reaction arising from the ventral wall of the tympanic bulla (c, arrow)

MRI was performed in 147 cats (75%), of which 102 had abnormal MRI findings. Abnormalities were identified in the middle and/or inner ear in 99 cats. Among the group of 99 cats with middle and/or inner ear abnormalities, vestibulocochlear and facial nerve enhancement were found in 18 and 14 cases, respectively (Figure 1). In three cats, vestibulocochlear nerve enhancement was the only MRI finding, and in 45 cats, there were no MRI abnormalities. CT was performed in 70 cases (35.7%), with 21 cats (10.7%) undergoing both diagnostic imaging modalities. CT abnormalities were found in 64 of 70 cats, all of them being related to the middle and/or inner ear (Figure 2). Otogenic intracranial extension from the OMI was observed in 26 cats (*n* = 20 on MRI and *n* = 6 on CT), and in two of these cats, adjacent intracranial empyema was found (the remaining 24 cats had meningeal enhancement only). Middle ear neoplasia was suspected in seven cases (*n* = 5 on CT, *n* = 1 on MRI, and *n* = 1 on both CT and MRI) and was subsequently confirmed by histopathology.

CSF analysis was performed in 74 cats and was abnormal in 14. In 11 of the 14 cats with abnormal results, the final diagnosis was OMI (six with signs of otogenic intracranial extension in MRI), and in three cats it was IVS. Mild to marked mixed pleocytosis was found in the 11 cats diagnosed with OMI (median 22 TNCC/µL, IQR 6‒610). Four of these cases had concurrent increased protein (median 1.2 g/L, IQR 0.69‒2.17). Rods and/or cocci were found in cytology for two of these cats. CSF culture was positive in two cases, and *Clostridium beijerinkii*, *Enterococcus faecalis* and *Staphylococcus epidermis* were isolated. Of the cats diagnosed with IVS, one had albuminocytological dissociation (protein 0.57 g/L) and two had mixed pleocytosis (21 and 10 TNCC/µL, respectively).

Forty‐nine cats within the OMI group (49/140, 68.6%) were diagnosed with aural polyps based on histopathology results, and in five other cats, a polyp was suspected based on imaging findings only. Fifty‐four cats had a positive bacterial culture of the material obtained via myringotomy or a surgical procedure. The most common bacterial isolates were *Staphylococcus* species (*n* = 31), *Pasteurella multocida* (*n* = 13), *Streptococcus* species (*n* = 5) and *Escherichia coli* (*n* = 4). Two cats had a positive culture for *Aspergillus*, and three cats had a positive culture for *Malassezia*.

Thirty‐eight cats (19.4%) received no treatment. Within this group, 34 had a final diagnosis of IVS, two were diagnosed with congenital vestibular syndrome, one died due to cardiopulmonary arrest under general anaesthesia during diagnostic tests for PVS and one was euthanased at the time of diagnosis owing to poor prognosis associated with the extension of a middle ear mass. Fine needle aspirate was non‐diagnostic in this case, and the owner elected euthanasia. Thirty‐one cats (15.8%) underwent solely medical management, which included anti‐nausea medication, oral or topical antimicrobial therapy, oral or topical glucocorticoids, antifungals, gabapentinoids, opioids, non‐steroidal anti‐inflammatory drugs, appetite stimulants and eye lubrication. Medical treatment was combined with surgery in 96 cats (49%) and with myringotomy and flushing of the tympanic cavity in 31 cats (15.8%). All cats that underwent surgery were diagnosed with OMI or neoplasia. Forty‐five cats with OMI secondary to aural polyps and 48 cats diagnosed with OMI alone were treated surgically. The most common surgical procedure was ventral bulla osteotomy (VBO), which was performed in 83 cats (bilateral in 12 cats), followed by total ear canal ablations with lateral bulla osteotomies in 10 cats. Traction avulsion of polyps alone was performed in three cats and as part of another surgical procedure in six cats. Regarding surgical complications, 70 cats developed Horner syndrome and 11 developed facial nerve paralysis. None of these cats had Horner syndrome or facial nerve paralysis preoperatively. Nine cats had temporary worsening of vestibular signs, one cat with previously diagnosed hypertrophic cardiomyopathy developed congestive heart failure and two cats suffered acute upper airway obstruction on recovery, one of which had to be mechanically ventilated.

Outcome data were obtained for 104 cats (Table 2). Of the 92 cats for which outcome could not be obtained, five were known to be dead at the time of data collection and 87 were lost to follow‐up. Short‐term outcome information only was available for 56 cats, with a median follow‐up time of 1 month (IQR 1‒2). Residual neurological deficits included head tilt (*n* = 35), ataxia (*n* = 16), Horner syndrome (*n* = 14), facial nerve paralysis (*n* = 2) and oscillating head movements (*n* = 1). Long‐term outcome information was available in 48 cats, with a median follow‐up time of 24 months (IQR 9.5‒46.5). In 10 cats, this was obtained by contacting the owners. Persistent neurological deficits included: head tilt in 19 cats and oscillating head movements in one, all of them diagnosed with OMI; ataxia in 12 cats (10 diagnosed with OMI, one with IVS and one with congenital vestibular syndrome); and Horner syndrome and facial nerve paralysis in six and five cats, respectively, all diagnosed with OMI. In the cats with known outcome, there was no significant difference in the extent of recovery between cats with OMI that had intracranial extension and those that did not (*p* = 0.057); in fact, all cats with intracranial extension showed some improvement (9/16) or resolution (7/16) of the clinical signs.

**TABLE 2:** Treatment modalities and associated short‐ and long‐term outcomes of 104 cats presented with peripheral vestibular syndrome

		Short‐term outcome			Long‐term outcome		
Diagnosis	Treatment	Full recovery	Partial recovery	No recovery	Full recovery	Partial recovery	No recovery
OMI without IC extension (*n* = 72)	Medical (*n* = 5)	‒	4	‒	1	‒	‒
	Myringotomy/ear flush (*n* = 20)	1	13	2	3	1	‒
	Surgical (*n* = 47)	4	18	2	9	14	‒
OMI with IC extension (*n* = 16)	Medical (*n* = 3)	‒	1	‒	‒	2	‒
	Surgical (*n* = 13)	1	1	‒	6	5	‒
IVS (*n* = 12)	No treatment (*n* = 12)	2	4	‒	5	1	‒
Neoplasia (*n* = 3)	Surgical (*n* = 3)	1	2	‒	‒	‒	‒
Congenital vestibular syndrome (*n* = 1)	No treatment (*n* = 1)	‒	‒	‒	‒	1	‒

Abbreviations: IC, intracranial; IVS, idiopathic vestibular syndrome; OMI, otitis media/interna.

Of the 104 cats with outcome data, 22 cats (21 initially diagnosed with OMI and one initially diagnosed with IVS) had at least one recurrence of their clinical signs. Recurrence occurred within 3 months of diagnosis in nine cats, between 3 and 6 months in eight cats and after 12 months in five cats. In two of these cats, recurrence occurred both within 3 months and after 12 months. Fourteen cats underwent advanced imaging on recurrence. Two of these cats were initially diagnosed and treated surgically for OMI (one with a polyp and one without), and no abnormalities were detected on imaging 48 and 30 months later. One cat originally diagnosed with OMI without a polyp experienced OMI in the contralateral ear, and 11 cats were diagnosed with recurrence of OMI in the same ear (five initially diagnosed with OMI alone and six with OMI secondary to a polyp), three of them with aural polyp regrowth. Seven cats with confirmed recurrence of OMI were initially treated surgically and four medically.

There was no correlation between the variables so all were included in the univariable analysis, which showed that age, centre, progression, history of otitis externa or upper respiratory signs, speed of onset of the clinical signs, duration of the clinical signs, progression or not of the clinical signs, presence of head tilt, nystagmus, ataxia, facial nerve paralysis, Horner syndrome, vestibulocochlear nerve MRI abnormalities and CSF abnormalities had some evidence of association (*p* < 0.25) with the final diagnosis (IVS or OMI). Following multivariable regression analysis, five factors remained statistically significant (Table 3). In this population, the likelihood of having a final diagnosis of OMI rather than IVS was five times higher in cats with a history of otitis externa or upper respiratory signs, six times higher in cats with facial nerve paralysis and 15 times higher in cats with Horner syndrome. Duration of clinical signs and age at diagnosis were also associated with the final diagnosis, with a 26% increase in the likelihood of having a final diagnosis of OMI per week of duration of the clinical signs and an 8% increase in the likelihood of having a final diagnosis of OMI per year of age.

**TABLE 3:** Final multivariable logistic regression model for risk factors associated with a final diagnosis of idiopathic vestibular disease versus otitis media/interna

	OR	95% CI	*p‐*value
Age (months)	0.993	0.986‒1.000	0.044
Duration of clinical signs (days)	1.034	1.008‒1.061	0.009
History of otitis externa or upper respiratory signs	5.245	1.849‒14.882	0.002
Facial nerve paralysis	6.531	1.287‒31.335	0.023
Horner syndrome	15.804	2.014‒124.02	0.009

Abbreviations: CI, confidence interval; OR, odds ratio.

## DISCUSSION

The most common cause of peripheral vestibular signs in the cats in the present study was OMI, followed by IVS. This supports recent findings where OMI was the most prevalent cause of PVS in a study of cats with peripheral and central vestibular dysfunction^9^ but differs from another study that also included both presentations in which IVS and OMI were equally represented.^7^ The latter study included cats undergoing MRI only, which could explain the difference in the results, as CT is more often performed in cases with high suspicion of OMI.^25^ In contrast, in dogs with peripheral vestibular dysfunction, IVS has been reported as the most prevalent condition.^31^, ^34^, ^35^

OMI in dogs typically results from otitis externa, while in cats, it most often occurs as a result of infection ascending through the Eustachian tube or secondary to an inflammatory aural polyp.^20^, ^26^, ^36^ More than one‐third of cats with OMI in the present patient cohort had a confirmed or suspected aural polyp. The prevalence of polyps in our population is higher than that reported in previous studies of feline PVS, which could be due to the high number of cats with histopathology of the tympanic bulla.^7^, ^9^ In agreement with previously published literature, the most common types of bacteria cultured from the middle ear were *Staphylococcus* species and *P. multocida*. ^20^, ^37^, ^38^, ^39^ Fungal infections were less frequent.^16^ Due to anatomical proximity, meningitis and OMI can occur concomitantly.^40^, ^41^ The perilymph contained within the osseous labyrinth of the inner ear communicates with the CSF in the subarachnoid space medial to the petrosal portion of the temporal bone via the perilymphatic duct.^2^ Meningeal enhancement has been reported to occur in approximately 50% of cats with PVS diagnosed with OMI.^11^ Meningoencephalomyelitis and intracranial abscesses have also been documented as a rare intracranial complication in cats.^13^, ^39^, ^41^, ^42^

The 26 cats with intracranial extension of OMI in our study had no evidence of CNS involvement on examination. CNS involvement in cats with signs that were localised to the peripheral vestibular system based on the neurological examination has been previously reported.^7^, ^9^, ^39^ In dogs, several studies have also reported that neurological examination findings are not always reliable for the localisation of vestibular dysfunction, with inflammation being the most frequent aetiology in incorrectly localised lesions.^34^, ^35^, ^43^ It is possible that treatment prior to referral reduces the severity of the neurological signs or that cats have delayed manifestation of central vestibular signs.^35^ The lack of difference in the degree of recovery between cats with OMI that had intracranial extension and those that did not suggests that cats with OMI can have a good outcome despite CNS involvement on advanced imaging.

CSF analysis is another diagnostic tool used to identify otogenic intracranial extension in cats.^11^ Five cats diagnosed with OMI that underwent MRI had mixed pleocytosis with no evidence of intracranial disease on imaging. An increased CSF cell count compared to a control group has been reported in cats diagnosed with OMI and no signs of CNS involvement.^23^ In another study, there was no association between abnormal CSF results and meningeal enhancement secondary to OMI.^11^ Only three cats with IVS had an abnormal CSF result, similar to findings described in dogs.^31^, ^44^, ^45^ CSF has not been shown to reliably differentiate between central and peripheral vestibular syndrome, and its analysis should therefore be interpreted in conjunction with the clinical presentation and other diagnostic tests.^44^ The specificities of elevated CSF TNCC and TP to differentiate between central and peripheral vestibular syndrome were 90% and 39%, respectively, in dogs.^44^

IVS was the second most common cause of PVS in our feline population. IVS is described as a peracute to acute non‐progressive condition with a good prognosis for spontaneous recovery.^3^, ^6^, ^46^ Although IVS can occur in cats of any age, in our study, the median age was 117 months, similar to the 108 months described by Grapes et al.^9^ These results are in contrast with those reported in a previous study, where the median age of cats with IVS was 62 months.^7^ Negrin et al. found that most cats with IVS presented with progressive clinical signs and associated this presentation with an atypical form of the disease.^7^ In our population, we found that most cats (83%) diagnosed with IVS had a non‐progressive or waxing and waning clinical course. The pathogenesis of IVS remains undetermined, and it is unclear if this disease represents a single condition or if there are multiple aetiologies involved in the different clinical courses. Infestation with botfly (*Cuterebra* species) larvae was suggested as a causative agent of a seasonal presentation of feline IVS in the United States, although no associated lesions have been found in histopathology, suggesting a possible role of environmental factors.^3^, ^8^, ^47^ Vestibulocochlear nerve enhancement was found in three cats with IVS, which has not been previously reported in cats, although vestibular and facial nerve enhancement has been reported in dogs diagnosed with IVS. In canine PVS, enhancement of these cranial nerves was more common than in cats, regardless of the underlying cause.^31^, ^45^ It is possible that due to the smaller cranial nerve diameter in cats, MRI may lack sensitivity for detecting subtle cranial nerve changes in this species. Alternatively, it may simply be that these nerves are not frequently affected in cats with PVS.

Approximately 20% of cats in our study had Horner syndrome. In agreement with previous reports in cats and dogs, the presence of Horner syndrome was associated with a diagnosis of OMI.^9^, ^34^ Horner syndrome in these cases is due to the interruption of the postganglionic oculosympathetic pathway. After leaving the cranial cervical ganglion, the postganglionic sympathetic fibres pass through the tympanic bulla and continue to the orbit to innervate the smooth muscle of the eyelid, periorbita and iris dilator muscle. It is therefore unsurprising that middle ear inflammation is frequently associated with Horner syndrome.^2^ Cats seem to be more susceptible to Horner syndrome than dogs^48^; Horner syndrome was present in 3.7‒16% of dogs diagnosed with PVS.^31^, ^34^ The incidence of Horner syndrome following bulla osteotomies is also significantly greater in cats compared to dogs.^49^, ^50^, ^51^, ^52^ This may be related to the differences in the neuroanatomical structures of the tympanic bulla between these species. The feline ventral tympanic bulla is divided into a smaller dorsolateral and a larger ventromedial compartment by an incomplete thin bony septum. Postganglionic sympathetic fibres extend across the promontory and continue into the dorsolateral compartment through the septum.^1^ Increased exposure or sensitivity of the postganglionic sympathetic fibres and the high prevalence of polyps that tend to severely affect the tympanic bulla are other possible reasons for the high frequency of Horner syndrome observed in cats.^50^, ^53^ In contrast, only one of the cats diagnosed with IVS had Horner syndrome. Although Horner syndrome has been previously reported in some canine and feline IVS patients, the cause for this is yet unknown.^9^, ^31^, ^34^, ^54^

Twenty‐six cats had facial nerve paralysis. The presence of facial nerve paralysis was significantly associated with a diagnosis of OMI, which differs from a previous study in which there was no association between facial nerve deficits and diagnosis.^9^ The facial nerve emerges from the cranial cavity together with the vestibulocochlear nerve through the internal acoustic meatus. It then courses through the facial canal in the petrosal portion of the temporal bone. Facial neuropathy due to middle ear disease is common due to this canal lacking a bony wall in one of its portions adjacent to the middle ear.^2^, ^55^ In the present study, the majority of cases with vestibulocochlear nerve changes and all cases with facial nerve abnormalities on MRI were diagnosed with OMI, likely reflecting an extension of the inflammatory process. Only two cats diagnosed with IVS had facial nerve paralysis on presentation. Conversely, facial nerve paralysis has been found to be present in half of dogs presenting with PVS and was associated with IVS in the majority of these.^31^, ^34^ Although concurrent facial neuropathy has been documented in 35‒67% of dogs with IVS,^31^, ^34^, ^35^, ^44^ this has only been reported previously in one cat.^26^ Possible explanations for this include anatomical variations between the two species or canine and feline IVS being different entities. Similarly to what was seen in our feline population, acute vestibular conditions in people are not typically associated with facial nerve paralysis. These conditions mainly include benign positional paroxysmal vertigo, Ménière's disease and acute vestibular neuritis.^56^, ^57^, ^58^ Fifteen percent of the human patients with idiopathic facial nerve paralysis or Bell's palsy have vestibular impairment only in the early stage.^59^, ^60^

Outcome information was obtained for 104 cats. Outcome data for cats with IVS and OMI were available in 12 and 88 cases, respectively. All cats with IVS showed partial or complete resolution of their clinical signs, while four cats with OMI did not improve. Persistent neurological deficits were also more common in patients diagnosed with OMI than in those with IVS. This differs from a previous study where the majority of cats with OMI recovered completely with medical treatment.^7^ Interestingly, none of the cats in our study that did not recover had otogenic intracranial extension, although five of them had recurrence of clinical signs. The outcome for cats with OMI and meningeal enhancement has been described to be poorer than for those without meningeal enhancement.^11^ Two cats included in our study had intracranial empyema; one recovered completely, while the other remained with a residual head tilt and facial nerve paralysis. Both cats had bulla osteotomies, and repeat imaging revealed that the intracranial lesions were resolving. Similarly, a previous study reported a better outcome in those cats with intracranial complications of OMI managed with VBO compared to the ones treated medically.^39^ Twenty‐one patients with OMI had recurrence of vestibular signs. Within the group of cats with confirmed OMI recurrence, seven had initial surgery and four were treated medically, with or without myringotomy/ear flush. Unfortunately, the small number of recorded cases undergoing further investigations at the time of relapse is too low to draw conclusions about the best treatment options for OMI causing PVS. In cats with IVS, seven of 12 had a complete recovery and only one had recurrence of episodes, similar to previous reports in the literature.^7^, ^32^ In canine IVS, relapses are more frequent and have been reported in 15‒20% of cases.^31^, ^45^

Only four cats died due to their PVS, and two were euthanased after a diagnosis of neoplasia. Ear canal tumours are more aggressive in cats than they are in dogs^10^, ^16^ Furthermore, neurological signs at the time of diagnosis have been associated with a poorer outcome in cats with tumours of the ear canal.^10^, ^61^ The most common persistent neurological deficit was head tilt, followed by ataxia. In dogs with PVS, head tilt and facial paresis are the most frequent persisting clinical signs, with a prevalence of approximately 30% for both neurological deficits.^31^

The limitations of the study were largely related to its retrospective nature and multicentre approach. There was no standardisation of the advanced imaging techniques, diagnostic tests or treatment methods used. The inclusion of cats that underwent solely CT may have overlooked inner ear and adjacent neural structure involvement, since MRI is preferable to CT for examining soft tissue components, including intralabyrinthine fluid.^23^, ^27^ This is of particular significance for the investigations performed to reach a diagnosis of IVS. However, only one cat diagnosed with IVS underwent CT. Another limitation was the inclusion of cats without histopathological diagnosis; this is relevant for the cats that could have been incorrectly classified as having OMI with polyps instead of neoplasia. Follow‐up information was unavailable in a large number of cases; therefore, outcome and recurrence data could not be included in the statistical analysis. There was a larger proportion of IVS cats lost to follow‐up compared to OMI cases, which may have led to bias in the interpretation of the outcome results. Furthermore, follow‐up information was often based on clinical records and telephone conversations. Without direct neurological examination, mild residual neurological deficits could have been underestimated. Finally, this study only included referral populations, and it can be argued that less severe presentations, such as IVS cases with rapid improvement in clinical signs, may not be referred.

In conclusion, OMI is the most common condition in cats diagnosed with PVS. There is an association between this condition and younger age, longer duration of clinical signs, history of otitis externa or upper respiratory signs, facial neuropathy and Horner syndrome, which could help clinicians in predicting the most likely diagnosis for cats with PVS. The majority of cats diagnosed with PVS have a complete or partial recovery, although residual neurological deficits are common. Recurrence of signs is more frequent in cats with OMI than in those diagnosed with IVS.

## AUTHOR CONTRIBUTIONS

*Conceptualisation*: Rita Gonçalves. *Data collection*: Jordina Caldero Carrete, Rita Gonçalves, Steven De Decker, Holger A. Volk, Rodrigo Gutierrez‐Quintana and Anna Morgana Mosel. *Methodology and formal analysis*: Jordina Caldero Carrete and Rita Gonçalves. *Writing original draft*: Jordina Caldero Carrete. *Writing—review and editing*: Jordina Caldero Carrete, Rita Gonçalves, Steven De Decker, Holger A. Volk, Rodrigo Gutierrez‐Quintana and Anna Morgana Mosel. All the authors have read and agreed to the published version of the manuscript.

## CONFLICT OF INTEREST STATEMENT

The authors declare no conflicts of interest.

## FUNDING INFORMATION

The authors received no specific funding for this work.

## ETHICS STATEMENT

Ethical approval for the use of the clinical data was granted by the Ethics Committee of the University of Liverpool.

## Data Availability

The data that support the findings of this study are available from the corresponding author upon reasonable request.
